# Perinatal programming of obesity: an introduction to the topic

**DOI:** 10.3389/fphys.2013.00255

**Published:** 2013-09-17

**Authors:** Catalina Pico, Andreu Palou

**Affiliations:** Laboratory of Molecular Biology, Nutrition and Biotechnology (Nutrigenomics), CIBER de Fisiopatología de la Obesidad y Nutrición (CIBEROBN), University of the Balearic Islands(UIB)Palma de Mallorca, Spain

**Keywords:** metabolic programming, maternal nutrition, gestation, lactation, obesity, leptin

A new paradigm for obesity prevention has emerged from the idea that nutritional and other environmental factors in early life have a profound influence on lifelong health. This notion is gaining increasingly great interest since the development of Barker's hypothesis of the “fetal origin of adult diseases” (Barker, 1990). A number of human epidemiology and animal model studies have shown that nutritional conditions during critical stages of development affect susceptibility to chronic diseases in adulthood, including cardiovascular disease, obesity, type II diabetes, and osteoporosis (Ong and Dunger, 2004; Remacle et al., 2004; Novak et al., 2006). Interactions between pre- and postnatal environment have also been described. For instance, accelerated postnatal growth after fetal growth restriction (the so-called “catch-up” growth) has been associated with adverse outcomes in later life (Eriksson et al., 1999; Dulloo et al., 2006).

The “thrifty *phenotype* hypothesis” (Hales and Barker, 2001) expresses that the rapidly changing incidence of obesity cannot be explained in terms of genetic changes. It proposes that when a fetus grows under conditions of undernutrition, various strategies in the development of organs and metabolic changes will undertake to maximize the chances of postnatal survival under conditions of food shortage; however, these adaptations will be detrimental to health if “adequate” nutrition is received later on (Hales and Barker, 2001). In this regard, the word “programming”, first introduced by Lucas (1991), has been adopted to describe the linkages between fetal life and long-term consequences (Gluckman and Hanson, 2004). It involves the notion that a stimulus that operates within a critical or sensitive period of development can lead to lasting or permanent effects on the structure or function of the body (Lucas, 2000).

Gestation and lactation are disclosed as critical periods, and both food restriction and overnutrition can lead to lasting effects in the offspring, thereby changing propensity to obesity and related metabolic alterations in adult life (McMillen and Robinson, 2005; Cottrell and Ozanne, 2008; Sanchez et al., 2012). Adaptive responses occurring in early life to face an adverse environment may result in new physiological set points intended to maximize immediate changes for survival. The “predictive adaptive response” hypothesis proposes that a mismatch between the pre- and postnatal environment is a major determinant of subsequent disease (Gluckman and Hanson, 2004).

Health or disease phenotypes are therefore the consequence of interaction between genetic and nutritional and environmental history, starting from the moment of conception, or even before. Within this context, here we describe, review and discuss known conditions in humans and animal models that have been associated with early programming of obesity, endocrine function and metabolic disorders, the mechanisms underpinning this linkage, and potential strategies and therapeutic targets for reversing it.

As far as contribution of the environment during perinatal life is concerned, maternal prenatal malnutrition has been associated with obesity and other metabolic alterations in the offspring in a large number of experimental and epidemiological observations (Ravelli et al., 1976; Vickers et al., 2000; Palou et al., 2010). The Dutch famine of 1944–1945 represents an emblematic example of later consequences of fetal malnutrition in humans (Ravelli et al., 1976). Studies performed in different animal models have provided direct proof of principle and biological plausibility of nutritional programming (Vickers et al., 2000; Ozanne, 2001; Langley-Evans, 2007; Thompson et al., 2007; Garcia et al., 2010, 2011; Palou et al., 2012). Unlike the fetal period, moderate maternal calorie restriction during lactation in rats exerts protective effects on the risk of obesity in adulthood (Palou et al., 2010, 2011). These aspects in perinatal maternal calorie restriction, and the different outcomes depending on gender and period, type and severity of restriction, as well as potential mechanisms involved in these processes have been reviewed (Pico et al., 2012).

Potential strategies and critical windows of development for reversing adverse effects induced as a consequence of developmental malprogramming have also been addressed (Vickers and Sloboda, 2012). In this regard, the hormone leptin is receiving special attention as a potential programming factor. The sources of leptin during early critical developmental windows include maternal transplacental transfer of leptin, endogenous fetal leptin, and also milk leptin during lactation (Palou and Pico, 2009; Vickers and Sloboda, 2012). Evidence in humans of the potential role of milk-borne maternal leptin during lactation comes from the observation of an indirect correlation between the concentration of leptin in breast milk and body weight increase in infants (Miralles et al., 2006; Doneray et al., 2009; Schuster et al., 2011). Experimentally, a cause-effect relationship of orally-taken leptin during early life in preventing obesity and other metabolic alterations in later life was first shown in neonatal rats supplemented with physiological doses of leptin throughout lactation (Pico et al., 2007). Moreover, subcutaneous injection of leptin into neonatal female rats born to undernourished mothers prevented the development of metabolic compromise in adulthood (Vickers et al., 2005). Therefore, leptin may represent an essential nutrient during lactation in protection against obesity and related disorders in later life, and may also be considered a strategy for the reversion of prenatal adaptations resulting from fetal undernutrition (Pico et al., 2011; Vickers and Sloboda, 2012).

The impact of maternal and paternal obesity and/or overnutrition on endocrine pancreatic development in the offspring and underlying mechanisms are also reviewed (O'Dowd and Stocker, 2013). The authors highlight that, in rodents, maintaining dams on a high-fat diet (HFD) for a single week during pregnancy can impair β-cell development and function in the offspring (Cerf et al., 2007) and that a paternal HFD also programs β-cell dysfunction (Ng et al., 2010); hence the combined impact of both maternal and paternal diet may produce an exacerbated phenotype.

Although little is known concerning the mechanisms involved, a review of several epidemiological and experimental studies endorses the association between maternal nicotine or tobacco exposure during gestation or lactation and the development of obesity and endocrine dysfunction (Lisboa et al., 2012). A smoke-free environment during the lactation period is essential for improving health outcomes and reducing the risk of future diseases.

Two original articles in rodents addressing the effects of maternal intake of an excess of carbohydrates during gestation in the offspring are also included (Beck et al., 2012; Samuelsson et al., 2013). Intake of a sucrose-rich diet by pregnant mice leads to an exacerbation of factors associated with cardiovascular risk in the adult offspring (Samuelsson et al., 2013). In turn, intake of carbohydrate supplements in rats during gestation appears to program insulin and leptin resistance in adult offspring and may predispose to the development of obesity under palatable energy dense diets (Beck et al., 2012). These associations remain to be explored in humans.
